# Construction Notes

**Published:** 1896-04-18

**Authors:** 


### CONSTRUCTION NOTES.

**New Dental Hospital of Ireland.**

The new Dental Hospital of Ireland was formally opened by the Right Hon. the Lord Mayor of Dublin. The site of the building is in Lincoln place. On the first floor are the laboratory and museum, on the second rooms for operations, the administration of anæsthetics, and for recovery therefrom. There is accommodation for the treatment of twenty patients in the principal room, which is to be devoted to minor operations. The second block of buildings, which is now to be worked on, is to include a lecture theatre and other departments, which will complete the establishment. The architect was R. Caulfield Orpen, Esq.; the contractor, Mr. John Good, of Dublin.

